# *Trichinella spiralis* Infection Mitigates Collagen-Induced Arthritis *via* Programmed Death 1-Mediated Immunomodulation

**DOI:** 10.3389/fimmu.2018.01566

**Published:** 2018-07-26

**Authors:** Yuli Cheng, Xing Zhu, Xiaohuan Wang, Qinghui Zhuang, Xu Huyan, Ximeng Sun, Jingjing Huang, Bin Zhan, Xinping Zhu

**Affiliations:** ^1^Department of Medical Microbiology and Parasitology, School of Basic Medical Sciences, Capital Medical University, Beijing, China; ^2^Department of Pediatrics, National School of Tropical Medicine, Baylor College of Medicine, Houston, TX, United States

**Keywords:** *Trichinella spiralis*, rheumatoid arthritis, programmed death 1, CD4^+^ T cell, immunomodulation

## Abstract

Helminth infection induces Th2-biased immune responses and inhibitory/regulatory pathways that minimize excessive inflammation to facilitate the chronic infection of helminth in the host and in the meantime, prevent host hypersensitivity from autoimmune or atopic diseases. However, the detailed molecular mechanisms behind modulation on inflammatory diseases are yet to be clarified. Programmed death 1 (PD-1) is one of the important inhibitory receptors involved in the balance of host immune responses during chronic infection. Here, we used the murine model to examine the role of PD-1 in CD4^+^ T cells in the effects of *Trichinella spiralis* infection on collagen-induced arthritis (CIA). Mice infected with *T. spiralis* demonstrated higher expression of PD-1 in the spleen CD4^+^ T cells than those without infection. Mice infected with *T. spiralis* 2 weeks prior to being immunized with type II collagen displayed lower arthritis incidence and significantly attenuated pathology of CIA compared with those of uninfected mice. The therapeutic effect of *T. spiralis* infection on CIA was reversed by blocking PD-1 with anti-PD-1 antibody, associated with enhanced Th1/Th17 pro-inflammatory responses and reduced Th2 responses. The role of PD-1 in regulating CD4^+^ T cell differentiation and proliferation during *T. spiralis* infection was further examined in PD-1 knockout (PD-1^−/−^) C57BL/6 J mice. Interestingly, *T. spiralis*-induced alteration of attenuated Th1 and enhanced Th2/regulatory T cell differentiation in wild-type (WT) mice was effectively diminished in PD-1^−/−^ mice characterized by recovered Th1 cytokine levels, reduced levels of Th2 and regulatory cytokines and CD4^+^CD25^+^Foxp3^+^ cells. Moreover, *T. spiralis*-induced CD4^+^ T cell proliferation suppression in WT mice was partially restored in PD-1^−/−^ mice. This study introduces the first evidence that PD-1 plays a critical role in helminth infection-attenuated CIA in a mouse model by regulating the CD4^+^ T cell function, which may provide the new insights into the mechanisms of helminth-induced immunomodulation of host autoimmunity.

## Introduction

After co-evolution with their hosts over a long period of time, helminths have developed the ability to induce host immune tolerance to facilitate their survival in the hosts. This helminth-induced immunomodulation may also benefit hosts to reduce pathological lesions caused by aberrant inflammatory responses that may underline many autoimmune disorders (1, 2). Rheumatoid arthritis (RA) is a chronic autoimmune disease characterized by synovial inflammation and bone erosion, which affects up to 1% of the population worldwide. There is strong evidence that abnormally activated Th1 and Th17 cells and impaired CD4^+^CD25^+^Foxp3^+^ regulatory T cell (Treg) contribute to the pathogenesis of RA (3). Helminth infections skew host immune response from Th1 to Th2/Treg characterized by stimulating the secretion of Th2 cytokine IL-4, IL-5, IL-10, and IL-13 (4) and induction of Treg development (5, 6). Th2 polarization and Treg-released IL-10, TGF-β downregulate the Th1 cell subset (6–8) that promotes the establishment of chronic infection (9). Immunomodulation by helminth infection has inspired to the idea of using helminthic therapy for atopic and autoimmune diseases in animal models and human trials which have provided convincing evidences for effectively alleviating a number of autoimmune diseases up to date (1). It has also been reported that helminth infection or helminth-derived products effectively alleviated the inflammatory arthritis by inducing Th2 responses or inducing Foxp3^+^ T regulatory cells (10, 11). However, the detailed molecular mechanisms behind the modulation of inflammatory diseases are yet to be clarified.

Programmed death 1 (PD-1) is a member of the B7 family on the surface of T cells that delivers inhibitory signals to promote self-tolerance by suppressing T cell inflammatory activity and reduce immune-mediated tissue damage (12). PD-1 is an important immune checkpoint to keep immune balance and exerts critical inhibitory functions in the setting of persistent antigenic stimulation such as during encounter of self-antigens, chronic infections, and tumors (13, 14). There is evidence supporting a distinct role of PD-1 and its ligands (PD-L1/B7-H1 and PD-L2/B7-DC) in regulating T cell tolerance and autoimmunity (15). In humans, a role for PD-1 in the regulation of self-tolerance and autoimmunity was suggested to be associated with autoimmune diseases such as systemic lupus erythematosus, RA, multiple sclerosis, and type 1 diabetes mellitus (16–19). In animal models of collagen-induced arthritis (CIA), defective expression of PD-1 has been confirmed to contribute to T cell hyperactivity within the inflamed joint (20). In human investigations, blockade of PD-1 with anti-PD-1 increases the risk of developing RA (21).

In recent years, many studies indicated that helminths may exploit the PD-1 pathway to modulate host immune system to minimize excessive inflammation and promote the chronicity of helminth infection (22–24). *Trichinella spiralis* is an intestine- and tissue-dwelled nematode that secretes molecules to modulate hosts’ immune system. Infection of this nematode or *Trichinella*-secreted proteins have been used for treatment of many hyperimmune-associated disorders in experimental studies such as asthma and allergic disorders (25), inflammatory bowel diseases (26, 27), encephalomyelitis (28), and type 1 diabetes (29), and significant alleviation of these diseases has been achieved.

It is well established that CD4^+^ T cells play a central role in the pathogenesis of RA (30). In this study, we aim to investigate whether *T. spiralis* infection affects the PD-1 expression in CD4^+^ T cells and its role in alleviation of arthritis using a CIA mouse model. We demonstrated for the first time that *T. spiralis* infection significantly alleviated CIA through activating the expression of PD-1 on CD4^+^ T cells. Moreover, this study highlights the importance of PD-1 as a checkpoint for *T. spiralis*-induced Th2 polarization and Treg generation which may provide new insights into the mechanisms of helminths’ immunomodulation on host autoimmunity.

## Materials and Methods

### Ethics Statement

This study was carried out in accordance with the recommendations of “IRB of Capital Medical University.” All animal experimental procedures were approved by the Animal Care and Use Committee of Capital Medical University (AEEI-2016-008) and comply with the National Institutes of Health Guidelines for the Care and Use of Experimental Animals.

### Mice

Male DBA/1 mice with 6–8 weeks old were purchased from the Laboratory Animal Services Center of Capital Medical University (Beijing, China) for induction of arthritis and related experiments. Wild-type (WT) and PD-1^−/−^ mice bred on the C57BL/6 background were purchased from the Jackson Laboratory (Stock no. 021157, USA). All mice were maintained under pathogen-free conditions with suitable humidity and temperature at the Animal Center of Capital Medical University.

### Helminth Infection Model

The *T*. *spiralis* (ISS 533) strain used in this study was maintained in female ICR mice. Mice were each infected with 400 infective *T. spiralis* muscle larvae by oral gavage.

### Induction of CIA

Experimental arthritis was induced in DBA/1 mice based on the method previously described (31). Bovine type II collagen (CII) purchased from Chondrex (Redmond, WA, USA) was dissolved in 0.01 M acetic acid at concentration of 2 mg/ml by stirring over night at 4°C and emulsified with the equal volume of complete Freund’s adjuvant. Male DBA/1 mice were immunized intradermally at the base of the tail with 0.1 ml emulsion containing 100 μg CII. The mice were boosted once with the same amount of CII emulsified with incomplete Freund’s adjuvant (Chondrex) 21 days after the first immunization. Induced arthritic mice were clinically assessed for redness and swelling of all limbs every other day up to 50 days. The clinical scores were assigned as previously described to evaluate disease (32) as follows: 0 = no signs of arthritis: 1 = swelling and/or redness of the paw or one digit; 2 = two joints involved; 3 = more than two joints involved and 4 = severe arthritis of the entire paw and digits. Each limb was graded, resulting in a maximal clinical score of 16 per animal.

### Histopathologic Analysis

The paws of the mice were removed after being euthanized and fixed overnight in 4% paraformaldehyde, decalcified in 20% EDTA for 6 weeks, and then dehydrated and embedded in paraffin. The tissue serial paraffin sections (2 mm) were cut along longitudinal axis, mounted and sections were stained with hematoxylin and eosin or toluidine blue (TB). The severity of inflammatory cell infiltration in joint and cartilage destruction was scored using a semi-quantitative scale described previously (33, 34). The severity of inflammatory cell infiltration was scored 0–4 as follows: 0 = no infiltrate; 1 = minimal (few cells in perisynovial and synovial tissues); 2 = mild (infiltrating cells more numerous in perisynovial and synovial tissues, and/or in bone marrow beneath joints); 3 = moderate (inflammatory cell infiltrate more intense in perisynovial and synovial tissues, and often extending into adjacent periosseous tissues and/or in bone marrow beneath joints); and 4 = marked (increasing intensity of inflammatory cell infiltrate in synovial and perisynovial tissues, and extending into adjacent periosseous tissues and/or widely dispersed in bone marrow). Cartilage damage was scored 0–5 according to the following criteria: 0 = normal; 1 = minimal (loss of TB staining only); 2 = mild (loss of TB staining and mild cartilage thinning); 3 = moderate (moderate diffuse or multifocal cartilage loss); 4 = marked (marked diffuse or multifocal cartilage loss); and 5 = severe (severe diffuse or multifocal cartilage loss).

### *In Vivo* Blockade of PD-1

In some experiments, the expression of PD-1 on immune cells in mice was blocked by injection of anti-mouse CD279 (PD-1) antibody (clone 29F.1A12, BioLegend, San Diego, CA, USA). Each mouse received 200 µg mAb intraperitoneally (i.p.) every 3 days, starting at 14 days post-infection until 3 days before the mice were sacrificed. For control mice, each was given the same amount of rat IgG2a isotype (clone RTK2758, BioLegend).

### Isolation of Lymphocytes From Spleen and Lymph Nodes

Four weeks after second immunization, the draining inguinal lymph nodes (ILNs) and spleens were removed and minced through a 70-µm cell strainer. Lymphocytes were isolated using Ficoll density-gradient centrifugation for flow cytometry or released-cytokine measurement.

### Spleen Cell Culture and Cytokine ELISA

Splenocytes were cultured in RPMI 1640 medium supplemented with 10% fetal bovine serum (FBS, Gibco, Grand Island, NY, USA), 100 U/ml penicillin, and 100 µg/ml streptomycin, at 2 × 10^6^ cells/ml in 24-well culture plates. During culture, the cells were stimulated with anti-CD3 (1 µg/ml)/anti-CD28 (1 µg/ml) (Peprotech, NJ, USA). The supernatants were collected at 48 h and kept frozen at −80°C until used. Cytokines IFN-γ, IL-4, IL-5, IL-13, IL-10, IL-17A, and TNF-α in the culture supernatants were measured with Ready-Set Go! Kits or recombinant cytokine/antibody sets from eBioscience (San Diego, CA, USA) according to the manufacturer’s instructions.

### Anti-CII Antibody Measurement

Sera were collected from mice 4 weeks after the second immunization of CII and the anti-CII-specific IgG and subtype IgG1 and IgG2a were measured by using antibody assay kit according to the manufacturer’s instructions (Chondrex, Redmond, WA, USA). Each sample was assayed in duplicate. OD values were measured at 490 nm using a Model 550 microplate reader and the results were analyzed using Microplate Manager III for Macintosh (Bio-Rad laboratories, Hercules, CA, USA).

### CD4^+^ T Cells Purification, CFSE Labeling, and Stimulation

CD4^+^ T cells were isolated from spleen or ILNs by positive selection using a magnetic-activated cell sorting system with anti-CD4 mAb (Miltenyi Biotec, Bergisch Gladbach, Germany) according to the manufacturer’s instructions. Isolated cells (5 × 10^6^ cells) were suspended in 1 ml sterile PBS containing 3% FBS. Carboxyfluorescein succinimidyl amino ester (CFSE, Invitrogen, Carlsbad, CA, USA) was added into the culture up to 5 µM for 5 min to label the cells. Labeling reaction was stopped by diluting with 9 ml of PBS containing 3% FCS and cells were washed twice. The labeled CD4^+^ T cells were used for the experiment of proliferation assessment and flow cytometry analysis. In experiment for determining non-specific T cell proliferation, the splenic CD4^+^ T cells isolated from *T. spiralis*-infected mice were cultured in plates coated with anti-CD3 (5 µg/ml, BioLegend, San Diego, CA, USA) in the presence of anti-CD28 (5 µg/ml, BioLegend) for 72 h. In experiment for determining specific anti-CII T cell proliferation, mice were immunized with CII on day ≥35 after *T. spiralis* infection. Then CD4^+^ T cells isolated from ILNs of mice on day 10 after CII immunization were cultured in the presence of CII for 72 h (20 µg/ml).

### Flow Cytometry

To analyze PD-1 expression in CD4^+^ T cells, the cells were stained with anti-mouse CD3-antigen-presenting cell (APC) (clone 17A2, eBioscience, Waltham, MA, USA), CD4-FITC (clone GK1.5, eBioscience), PD-1-PE (clone J43, eBioscience). To detect intracellular cytokine expression, T cells from each mouse were stimulated with 2 µl/ml. Cell Activation Cocktail (with Brefeldin) (BioLegend, San Diego, CA, USA) in complete RPMI 1640 medium for 6 h at 37°C in 5% CO_2_, then collected and surface stained with CD3 and CD4. The cells were washed, fixed, and permeabilized with cytofix/cytoperm buffer (BD Pharmingen), then intracellularly stained with anti-IFN-γ PE-Cyanine7 (clone XMG1.2), IL-4 PE-Cyanine7 (clone 11B11), and IL-17A PE-Cyanine7 (clone eBio17B7), or rat IgG1 and IgG2a isotype antibody (clone eBRG1, all from eBioscience) as control, respectively. To determine Tregs, the CD4^+^ cells were surface stained with anti-mouse CD3-PerCP (clone 17A2), CD4-FITC (clone RM4-5), and CD25-APC (clone PC61.5) in a Mouse Regulatory T cell Staining Kit (eBioscience). The cells were then permeabilized with cold Fix/Perm Buffer, and stained with anti-mouse Foxp3-PE (clone FJK-16s) or rat IgG2a isotype control antibody (clone eBR2a). Following immunofluorescence staining, samples were analyzed on a Flow cytometer (BD) using flowjo software (TreeStar). The cells were gated on CD3^+^ CD4^+^ T cells.

### Statistical Analysis

Statistical analysis was performed using SPSS version 11.0. All data are expressed as mean ± SEM. To determine differences between multiple groups, analysis of variance was used with *post hoc* comparisons using Tukey’s method. For comparison between two groups, a Student’s *t*-test was performed. A *P*-value <0.05 was considered significant.

## Results

### *T. spiralis* Infection Upregulates PD-1 Expression in CD4^+^ T Cells

**Figure 1:**
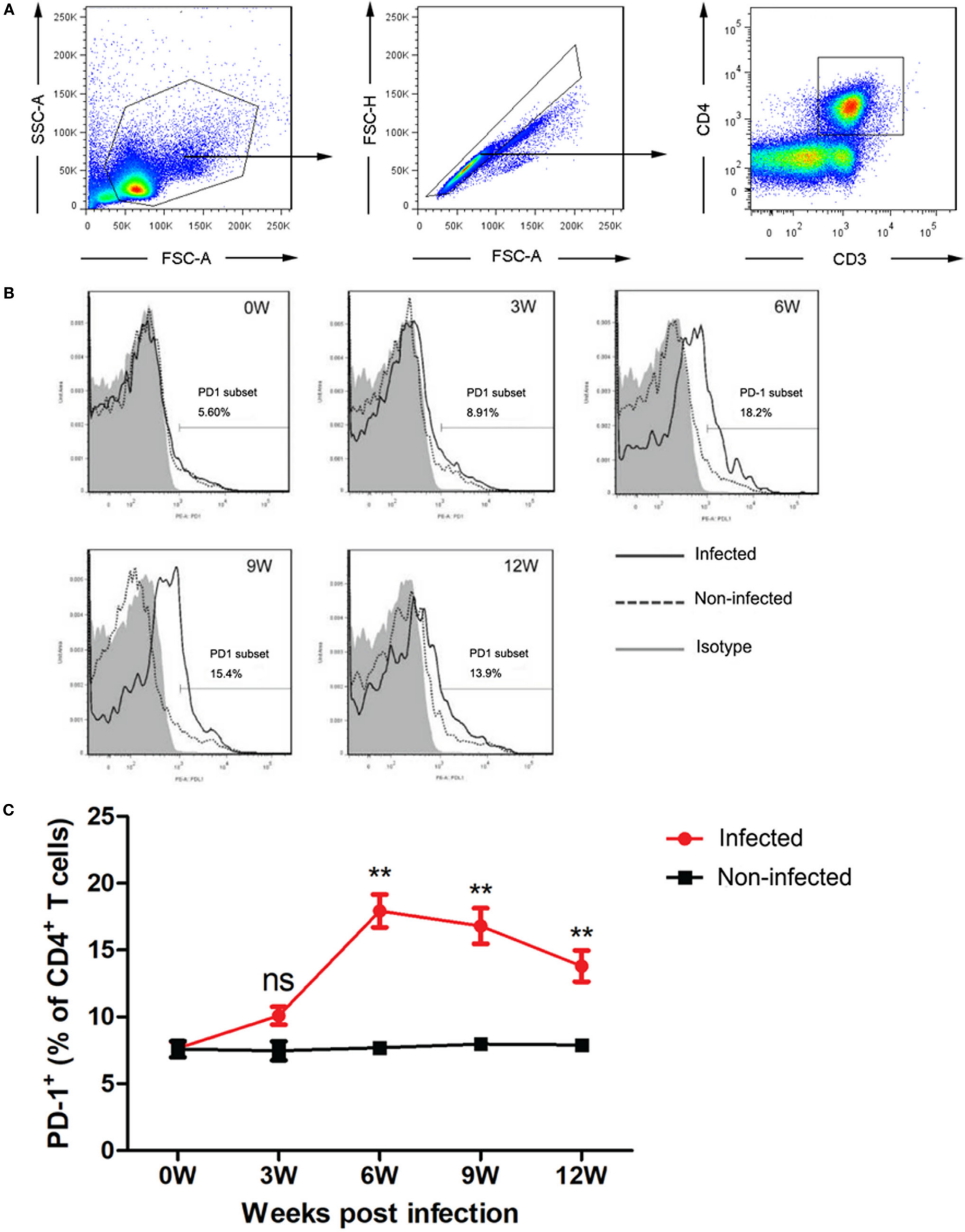
Dynamics of programmed death 1 (PD-1) expression in CD4^+^ T cells from *Trichinella spiralis*-infected mice. **(A)** FACS gating strategy for CD4^+^ T cells expressing PD-1. PD-1 gating was shown based on the PD-1 isotype control. **(B)** Flow cytometry showing the PD-1 subset in CD4^+^ T cells from spleen of infected mice compared with those from non-infected mice. Non-specific isotype antibody was used as control. The representative PD-1 expression is represented in solid line in *T. spiralis*-infected mice and in dotted line in non-infected mice. Isotype control is illustrated in gray. **(C)** The dynamic expression of PD-1 in CD4^+^ T cells of mice during *T. spiralis* infection. Data are expressed as mean ± SEM from three independent experiments (*n* = 5 mice per group).

Programmed death 1 expression in spleen CD4^+^ T cells was upregulated in mice infected with *T. spiralis*. Increased expression of PD-1 in spleen CD4^+^ T cells was observed in the initial acute stage of infection, peaked at week 6, and followed by a slight decline thereafter (Figure 1) with significant difference to the baseline expression level in the CD4^+^ T cells of normal mice.

### *T. spiralis* Infection Alleviates CIA Through PD-1 Pathway

**Figure 2:**
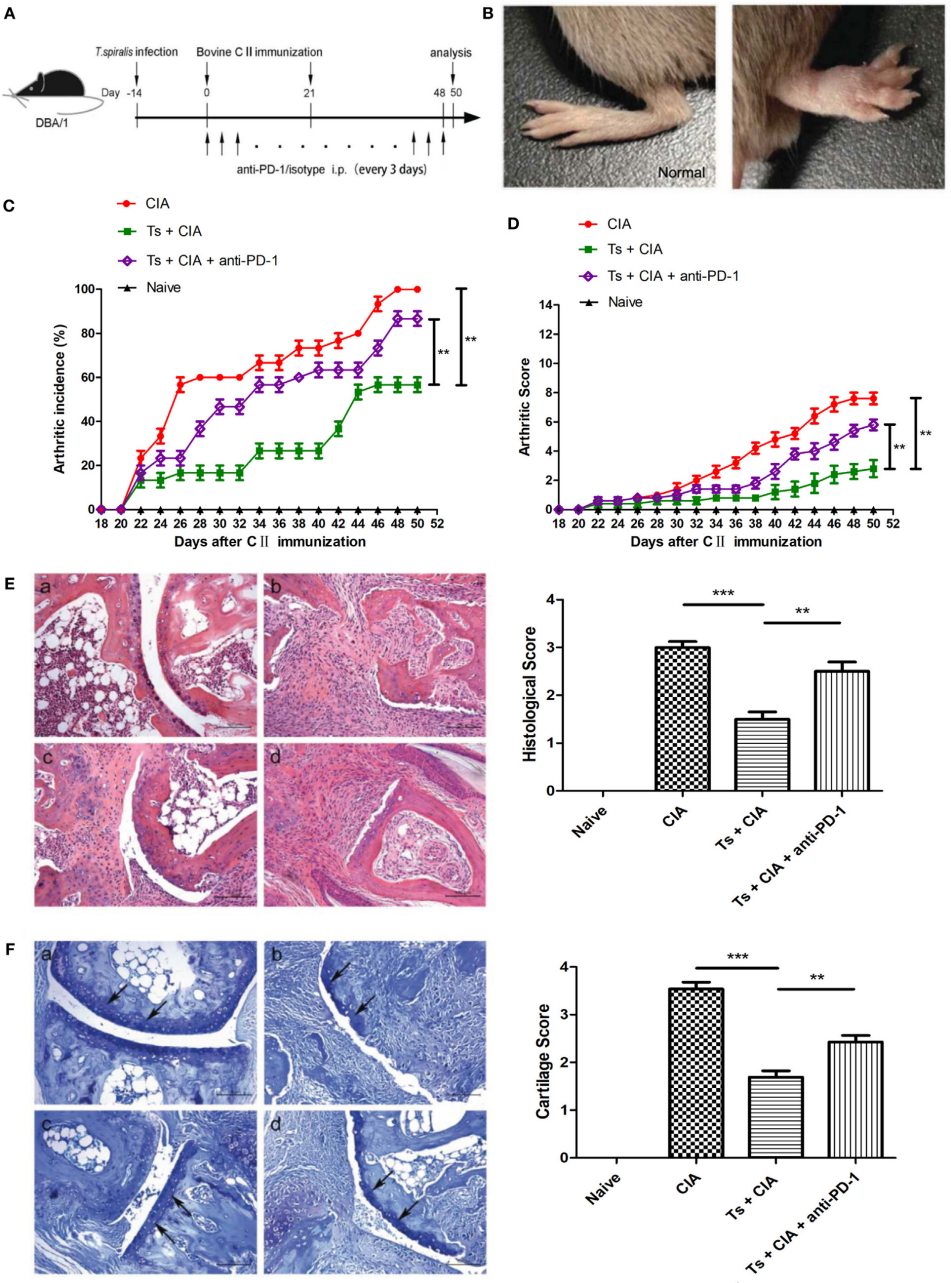
Programmed death 1 (PD-1) blockade abated *Trichinella spiralis* infection-induced attenuation of collagen-induced arthritis (CIA) in DBA/1 mice. **(A)** The regimen of study including the induction of CIA, treatment with infection of *T. spiralis* and anti-PD-1 antibody. **(B)** Hind paw of a mouse before and after induction of arthritis with bovine type II collagen (CII). Arthritic incidence **(C)** and total arthritic score **(D)** of mice from different groups (*n* = 10) at different time points. **(E)** Hematoxylin and eosin of the representative inflamed joints in the hind paw of mice from different groups at day 50 post first CII immunization, the histological score was shown on the right (mean ± SEM, *n* = 6). **(E)** The toluidine blue staining and cartilage score **(F)** of the representative inflamed joints in the hind paw of mice from different groups at day 50 post first CII immunization. (a) Naïve untreated control, (b) CIA, (c) Ts + CIA, (d) Ts + CIA + anti-PD-1. The bar graphs on the right side **(E,F)** show the histopathological scores for each group given as mean ± SEM (*n* = 6 mice per group). Statistical significance is determined by Student’s *t*-test for single comparison. **P < 0.01 and ***P < 0.001 (one-way analysis of variance).

To determine whether the infection of *T. spiralis* alleviates the severity of CIA in mice, mice were infected with *T. spiralis* 14 days prior to the first immunization of CII. The representative paw of mice with CIA was shown in Figures 2A,B. As shown in Figures 2C,D, *T. spiralis*-infected CIA mice displayed significant reduction in the incidence of induced CIA and alleviated arthritic score compared with uninfected CIA mice. PD-1 blockade with specific antibody significantly increased the incidence and severity of arthritis in *T. spiralis*-infected CIA mice (Figures 2C,D). Histologic analysis of the paws showed significantly decreased inflammation scores and cartilage destruction in *T. spiralis*-infected CIA mice compared with non-infected CIA mice. Similarly, the amelioration of inflammatory cell infiltration and cartilage destruction in *T. spiralis*-infected CIA mice was effectively reversed by the blockage of PD-1 with anti-PD-1 (Figures 2E,F). Isotype IgG2a control had no any effect on CIA (data not shown). These data suggested that PD-1 plays an important role in the inhibitory effect of *T. spiralis* infection on CIA in mouse.

### Nematode-Induced Inhibition of Th1/Th17 Responses and Enhancement of Th2 Responses Were Abated by Blocking PD-1 in CIA Mice

**Figure 3:**
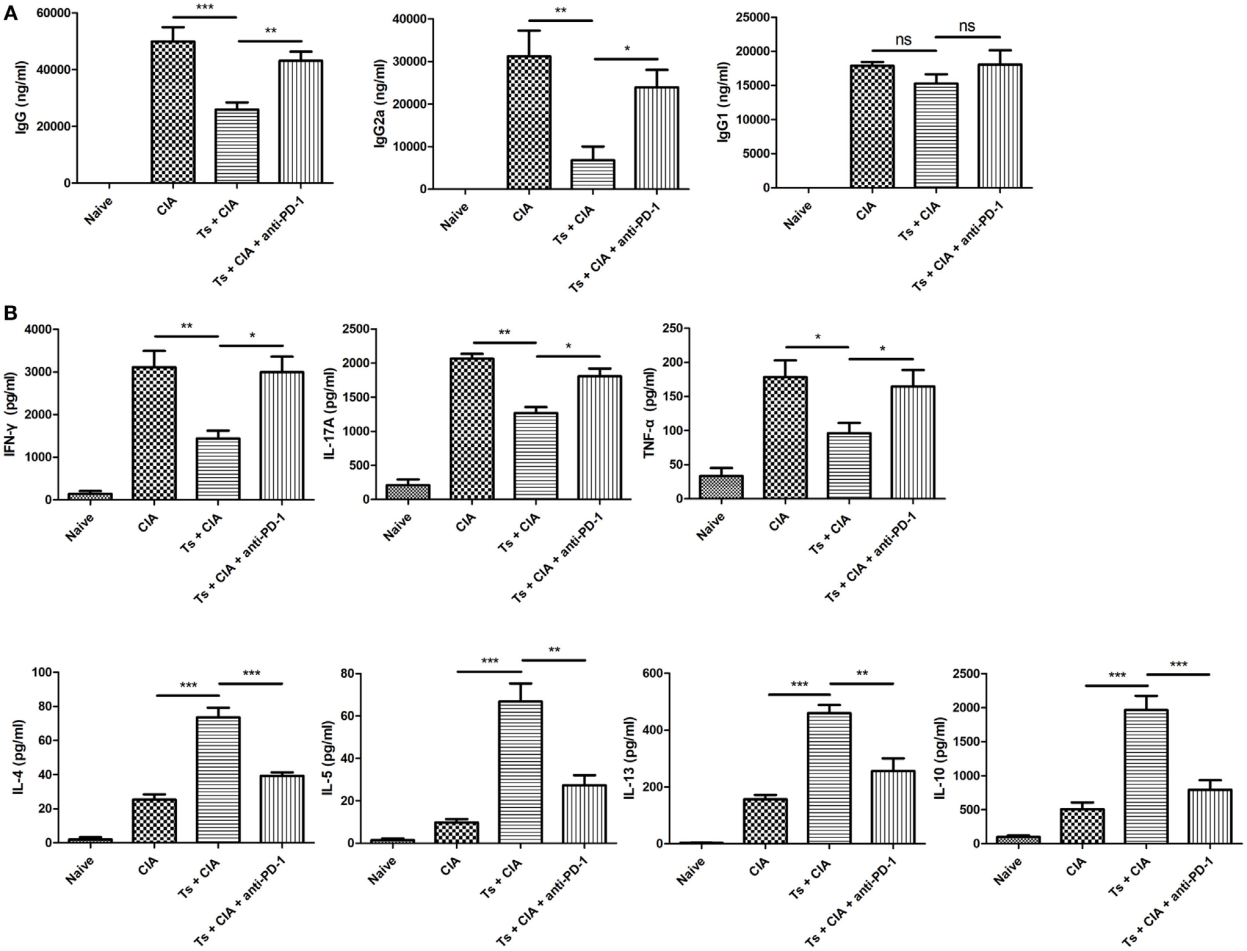
Programmed death 1 (PD-1) blockade offsets the inhibited Th1/Th17 responses and enhanced Th2 responses induced by *Trichinella spiralis* infection in collagen-induced arthritis (CIA) mice. Anti-type II collagen (CII) antibody and cytokine profile were determined by ELISA on day 50 after immunization. **(A)** Serological titers of anti-CII total IgG and subtypes IgG1 and IgG2a in mice of different groups. **(B)** Cytokine profiles secreted by splenocytes stimulated with anti-CD3 (1 μg/ml)/anti-CD28 (1 µg/ml) for 48 h. Data are expressed as mean ± SEM for duplicate serum samples or cell cultures. Data are representative of results from two independent experiments (*n* = 6 mice per group). *P < 0.05; **P < 0.01; ***P < 0.001; ns, not significant (one-way analysis of variance).

To understand the mechanisms involved in the *T. spiralis* infection-attenuated CIA, the humoral and cellular immune responses were measured in the treated mice. It is well established that anti-CII antibody is involved in the pathogenesis of CIA (35). Serological levels of antigen-specific total IgG, and subtypes IgG2a and IgG1 were measured. As shown in Figure 3A, the anti-CII total IgG level in the sera of mice infected with *T. spiralis* was significantly lower than that in mice without infection. Subtype analysis demonstrated that the reduced IgG level mostly resulted from the reduction in the IgG2a (Th1) but not in IgG1 (Th2). The reduced levels of IgG and IgG2a in *T. spiralis*-infected mice were effectively restored when PD-1 was blocked using anti-PD-1 antibody. The cytokine profile of splenocytes stimulated by anti-CD3/anti-CD28 antibodies showed that *T. spiralis*-infected CIA mice produced significantly lower levels of pro-inflammatory cytokines including IFN-γ (Th1), IL-17 (Th17), and TNF-α, but higher level of Th2 cytokines IL-4, IL-5, IL-13, and regulatory cytokine IL-10 compared with CIA mice without infection (Figure 3B). However, the nematode-reduced pro-inflammatory cytokines and boosted Th2 cytokines in CIA mice were significantly abated when PD-1 was blocked using anti-PD-1 antibody. The above results indicate that alleviation of CIA by the infection of *T. spiralis* is associated with the reduced Th1/Th17, enhanced Th2 responses possibly through stimulating the expression of suppressive PD-1 in the immune cells.

### PD-1 Knockout Offsets *T. spiralis*-Induced Anti-Inflammatory Modulation of CD4^+^ T Cells

**Figure 4:**
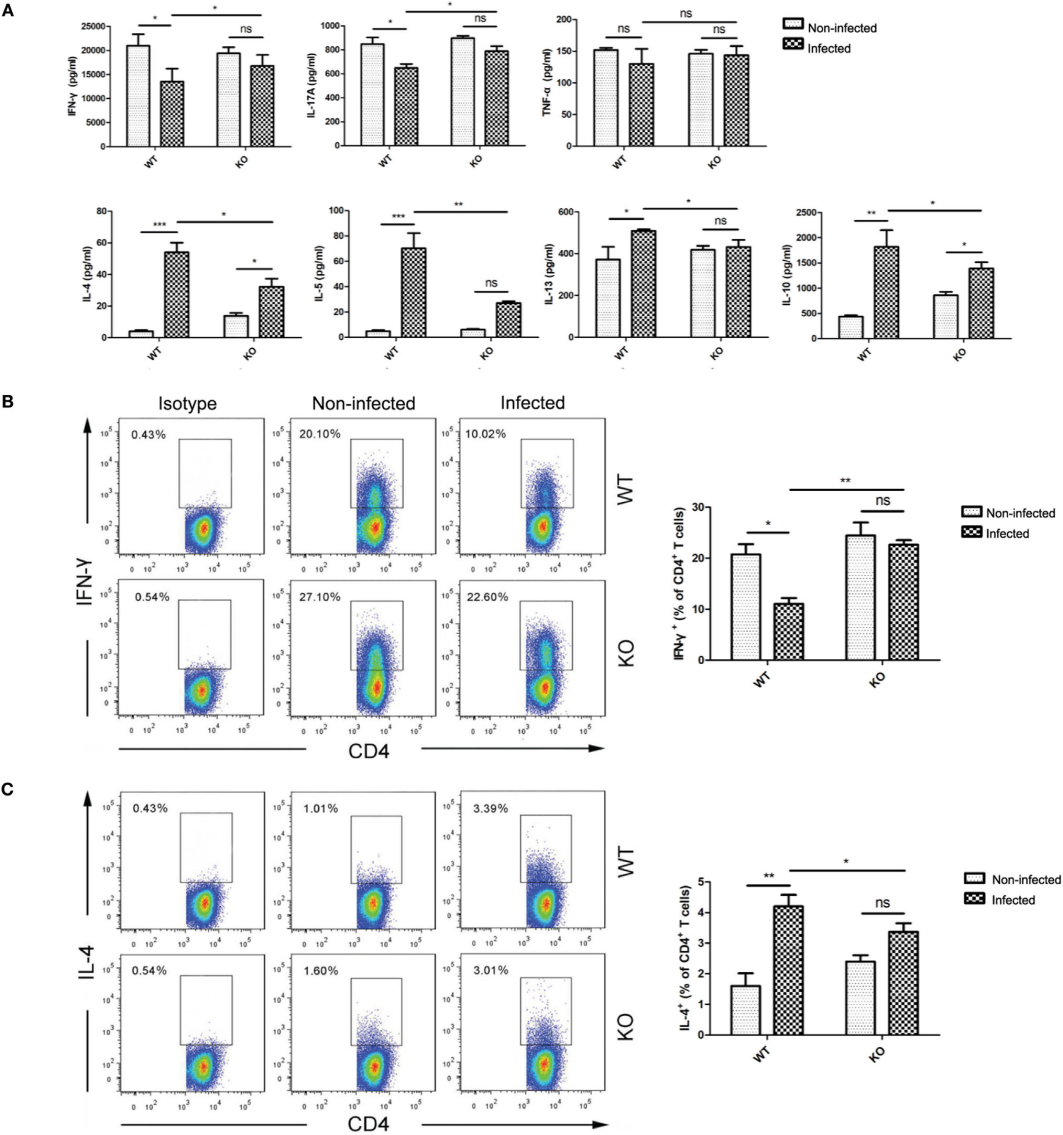

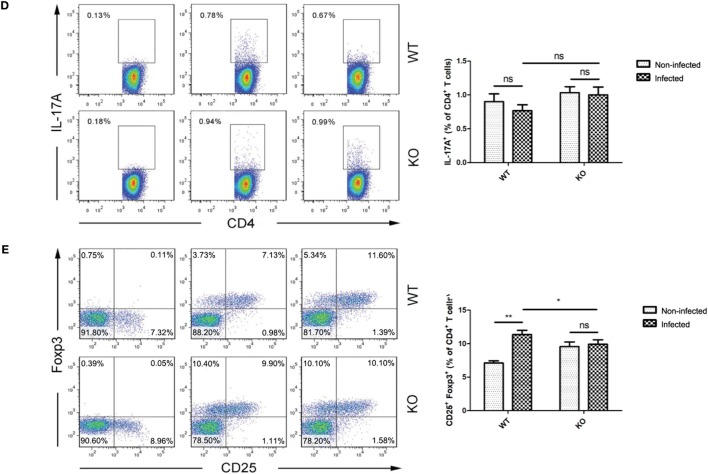
Programmed death 1 (PD-1) knockout abated *Trichinella spiralis*-induced Th1/17 inhibition and Th2/regulatory T cell (Treg) enhancement. Single-cell suspensions of spleens from wild-type (WT) and PD-1^−/−^ mice (KO) infected with or without *T. spiralis* were prepared. **(A)** Cytokine profiles secreted by splenocytes (IFN-γ, IL-17A, TNF-α, IL-10, IL-4, IL-5, and IL-13) upon stimulation with anti-CD3/CD28 for 48 h measured by ELISA. **(B–E)** Representative staining and percentages for IFN-γ-, IL-4-, IL-17-producing CD4^+^ T cells and CD4^+^ CD25^+^ Foxp3^+^ Treg cells by FACS. Data are expressed as mean ± SEM from three independent experiments (*n* = 5 mice per group). *P < 0.05; **P < 0.01; ns, not significant (paired Student’s *t*-test).

To further investigate whether *T. spiralis* infection-induced immunomodulation is PD-1 mediated, we profiled cytokines secreted by splenocytes upon stimulation of anti-CD3/anti-CD28 in WT and PD-1^−/−^ C57BL/6 J mice infected with or without *T. spiralis*. As shown in Figure 4A, the inhibited IFN-γ and IL-17 production following *T. spiralis* infection in WT mice was recovered in PD-1^−/−^ mice. By contrast, *T. spiralis*-enhanced IL-4, IL-5, IL-13, and IL-10 production in WT mice was abated in PD-1^−/−^ mice. This result further suggests that *T. spiralis* may activate PD-1 pathway to inhibit Th1- and Th17-associated pro-inflammatory cytokine production and to boost Th2-associated anti-inflammatory cytokine and regulatory cytokine production. Flow cytometry also showed that *T. spiralis* infection decreased IFN-γ^+^ (Th1), and increased IL-4^+^ (Th2) CD4^+^ T cells and CD25^+^Foxp3^+^ Tregs, but little effected on IL-17A^+^ (Th17) CD4^+^ T cells. However, these *T. spiralis*-induced attenuated Th1 and enhanced Th2/Treg differentiation in WT mice were effectively diminished in PD-1^−/−^ mice (Figures 4B–E). These results with PD-1^−/−^ mice further confirm that *T. spiralis*-induced differential control of CD4^+^ T cell subsets is PD-1 mediated, suggesting that PD-1 play a critical role in *T. spiralis*-induced immunomodulation.

**Figure 5:**
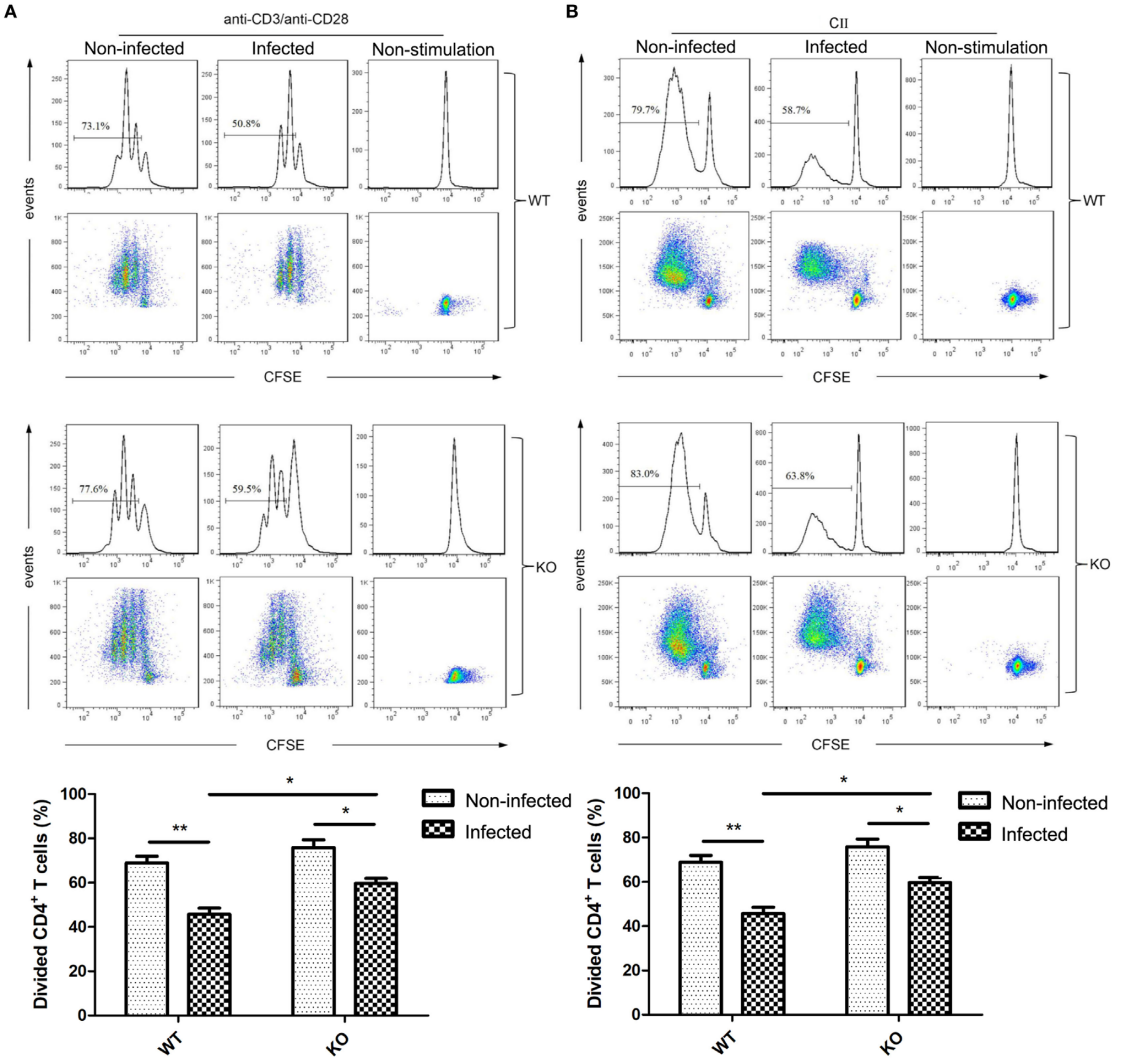
Programmed death 1 (PD-1) knockout partially restored suppressed proliferation of CD4^+^ T cells in *Trichinella spiralis*-infected mice. CD4^+^ T cells were isolated from C57BL/6 wild-type (WT) and PD-1^−/−^ mice and stained with CFSE. Decay of CFSE staining of CD4^+^ T cells was determined by flow cytometry. **(A)** FACS analysis of CD4^+^ T cell proliferation in response to non-specific stimulation. Purified CD4^+^ T cells from spleen of uninfected or *T. spiralis*-infected mice at day ≥35 of infection were stained with CFSE and incubated in the presence of anti-CD3/anti-CD28 for 72 h. **(B)** FACS analysis of CD4^+^ T cells proliferation in response to type II collagen (CII)-specific stimulation. Mice with or without *T. spiralis* infection (at day ≥35 of infection) were immunized with CII. CD4^+^ T cells were purified from LNCs of mice at day 10 post CII immunization and stained with CFSE, then incubated in the presence of CII (10 µg/ml) for 72 h. Irradiated naïve splenocytes were used as antigen-presenting cells (APCs). Data were expressed as mean ± SEM from two independent experiments (*n* ≥ 3 mice per group). **P < 0.05; *P < 0.01 (paired Student’s *t*-test).

To determine the responsiveness of T cell in *T. spiralis*-infected mice, we examined the CD4^+^ T cell proliferation upon non-specific (anti-CD3/anti-CD28) and antigen-specific stimulation in WT and PD-1^−/−^ mice with or without infection. The proliferation of splenic CD4^+^ T cells upon non-specific stimulation (anti-CD3/anti-CD28) was significantly inhibited in cells from *T. spiralis*-infected mice compared to those from non-infected mice. The inhibited CD4^+^ T cell proliferation was partially restored in mice with PD-1 knockout (Figure 5A). We further analyzed the antigen-specific T cell proliferation in CD4^+^ T cells isolated from ILNs of CII-immunized mice upon CII stimulation. Similarly, the inhibition of CD4^+^ T-cell proliferation upon re-stimulation of specific antigen CII in *T. spiralis*-infected mice was partially lifted in T cells from PD-1^−/−^ mice (Figure 5B). These results suggest that PD-1 partially contributes to *T. spiralis*-induced hyposensitivity of CD4^+^ T cells.

## Discussion

Immune responses are regulated by the balance of positive and negative regulatory pathways. Negative regulatory pathways are crucial for peripheral self-tolerance and preventing autoimmunity, and can function through signals delivered by cell surface inhibitory receptors, immunoregulatory cytokines, and Tregs (30). Multiple co-inhibitory receptors such as lymphocyte activation gene 3 (LAG-3), B- and T-lymphocyte attenuator 4 (BTLA-4), cytotoxic T-lymphocyte antigen 4 (CTLA-4), and T cell membrane protein 3 (Tim-3), CD244, and CD160 are expressed in T cells to dampen immune activation and limit immune-mediated pathology (36, 37). Recent studies demonstrated that these inhibitory receptors also play an important role in the response to pathogens. It is reported that helminth infection drives the sustained expression of T cell inhibitory receptors, which may negatively regulate proliferation and the production of pro-inflammatory cytokines by helminth antigen-specific T cells (38–40). Because these molecules largely function to prevent over exuberant T cell activation, their essential role in preventing parasite-induced immunopathology have been confirmed in animal studies (38, 41). However, the impact of these parasite-induced inhibitory molecules on autoimmune pathology has not been clarified.

Programmed death 1 plays a critical role in maintaining host immune homeostasis during chronic infection (42, 43). In this study, we observed upregulation of PD-1 in lymphocytes of mice infected with *T. spiralis*. The upregulation of PD-1 was also observed in the chronic infections of *Schistosoma japonicum* (24), *Fasciola hepatica* (44), *Taenia solium* (45), *Echinococcus multilocularis* (45) related to the survival of helminth in the host and reducing infection caused immunopathology.

To determine if the *T. spiralis* infection reduces the pathology of inflammatory arthritis, we established a collagen-induced mouse model (CIA). The CII-reactive CD4^+^ T cells are the primary mediators of disease induction by driving autoantibody production in B cells and enhancing the chronic inflammatory response (46, 47). Our results demonstrated that *T. spiralis* infection significantly mitigated the pathology of CIA in mice mostly through reducing Th1/Th17 pro-inflammatory responses and boosting Th2 response. The results are in accordance with previous studies that showed increased Th1/Th17 cellular response played a key role in the CIA (48, 49). *T. spiralis* infection reduces these pro-inflammatory responses therefore alleviates pathology of CIA. It is well known that helminth chronic infection-induced Th2 polarization is also involved in the therapeutic effects on autoimmune diseases. *Nippostrongylus brasiliensi*-induced activation of Th2 axis effectively mitigates the course of inflammatory arthritis and this protective effect is dependent on IL-4/IL-13-induced STAT6 pathway (50). *F. hepatica* excretory–secretory products were reported to protect against experimental autoimmune encephalomyelitis *via* type 2 cytokines (51).

Given that the PD-1 expression is upregulated in the CD4^+^ T cells of *T. spiralis*-infected mice and PD-1 is an important inhibitory and checkpoint receptor on immune cells, we postulated that *T. spiralis*-induced PD-1 expression may be involved in the alleviation of CIA by suppressing Th1 and Th17 responses and boosting Th2 response. Indeed, we observed that the reduced pathology of CIA in *T. spiralis*-infected mice was correlated with the increased expression of PD-1 in CD4^+^ T cells. Blocking PD-1 with anti-PD-1 mAb seriously reversed the amelioration of CIA in *T. spiralis*-infected mice, correlating with recovered level of Th1/Th17 response and reduced Th2 response. PD-1 knockout also demonstrated its reversion to *T. spiralis* infection-involved Th1 and Th2 changes, however, it did not change much the frequency of Th17 within CD4^+^ T cells at day 42 post-infection (Figure 4D), possibly because the stage of chronic *T. spiralis* infection may not affect much on IL-17 expression (52). Th17 cells are known to be involved in the inflammatory immune responses and autoimmune diseases as shown in CIA induction in this study (Figure 3B). However, it is not well understood the role of Th17 cells in the helminth infections (53), even though it has been observed that *T. spiralis* infection really reduced the CIA-induced Th17 secretion (Figure 3B).

Our results provide strong evidences at the first time that PD-1 pathway is involved in immunomodulation induced by *T. spiralis* infection that attenuates autoimmune-related arthritis. We postulate that pre-infection with *T. spiralis* may induce an anti-inflammatory modulation ahead of the initiation of CIA *via* activating the PD-1 pathway.

The costimulatory pathway consists of the PD-1 and its ligands, PD-L1 and PD-L2, delivering inhibitory signals that regulate the balance among T-cell activation and immune-mediated tissue damage to prevent autoimmunity (13, 54, 55). Manipulation of PD-1:PD-L1/2 pathway is considered a potential therapeutic approach for treating autoimmune diseases (15). Impact of PD-L:PD-1 axis on differentiation of CD4^+^ T cell subsets has been reported in previous studies (32, 56, 57). In this study, we also observed that the increased expression of PD-1 in CD4^+^ T cells in *T. spiralis*-infected mice and knockout of PD-1 resulted in the recovery of inhibited CD4^+^ T cell proliferation caused by nematode infection, indicating PD-1 is involved in the nematode infection caused regulation of CD4^+^ T cells. At the meantime, we identified that the Th2 polarization and Treg generation induced by *T. spiralis* infection were effectively diminished in PD-1^−/−^ mice. The results imply a critical role of PD-1 in modulating the balance of Th1/Th2 and Treg responses upon infection of *T. spiralis* that may outline the molecular mechanism behind the helminth-induced immunomodulation. Activation of PD-L1:PD-1 pathway may result in the enhanced Foxp3 expression and suppressive function of established induced regulatory T (iTreg) cells (12). CD4^+^CD25^+^FoxP3^+^ Tregs are highly involved in the regulation of immune responses and preventing autoimmunity (58–60). *Schistosoma mansoni* and *T. spiralis* derived antigens have been demonstrated to exert protective effect against adjuvant arthritis by upregulation of the Foxp3^+^ Tregs (10). Here, we confirmed that *T. spiralis*-induced expression of Foxp3 is highly dependent on PD-1 expression on immune cells, which implies that PD-1-mediated generation of Foxp3^+^ Tregs may contribute to the *T. spiralis*-attenuated CIA. However, different helminth infection may modulate host immune regulation through different PD-L/PD-1 pathway. The conditional deletion of PD-L1 impaired Th2 polarization and cytokine production in mice following *N. brasiliensis* infection (56). By contrast, blockade of PD-1 results in recovery of hyporesponsive Th2 cell function which was mediated through PD-L2 during chronic infection with *Litomosoides sigmodontis* (22). While the reasons for the discrepancies regarding the role of PD-1 in regulating Th2 cytokine production remain unclear, it seems to be related to the types of PD-L which interact with PD-1 expressed in the CD4^+^ T cells to control the function of Th subsets. It has been demonstrated that PD-L1 and PD-L2 have distinct roles in regulating host Th cell differentiation in response to leishmaniasis (61). Moreover, PD-1 has been suggested to enhance Th2 responses under conditions of sub-optimal TCR stimulation, which might be associated with the type of antigen (62).

In addition to activating Th2 cell-biased responses, helminths have also developed multiple mechanisms to regulate the host immune system. Humans with chronic infectious diseases, including helminth infection, experience sustained immune activation that is often accompanied by T cell hyporesponsiveness. Recent studies revealed that helminth infection induced T cell hyporesposiveness might contribute to suppression of autoimmune diseases. For example, infection with *Schistosome* regulates lymphocyte function *in vivo* by suppressing T cell activation (63, 64). Since PD-1 is described as a co-inhibitory receptor which induces T cell exhaustion, we examined the role of PD-1 in regulating T cell proliferation in *T. spiralis*-infected mice. Our study demonstrated a decreased T cell proliferation in *T. spiralis*-infected mice in response to both non-specific and CII-specific stimulation. However, PD-1 deletion only partially restored *T. spiralis*-suppressed CD4^+^ T cells proliferation. Although blockage of PD-1 can reverse the hyporesponsiveness to *S. japonicum* (24) and *L. sigmodontis* (22), many possible mechanisms may underlie the incomplete recovery of the suppressed T-cell proliferation induced by *T. spiralis* infection after PD-1 deletion observed in this study. Helminth infection modulates host T cell function through multiple factors including induction of Tregs, IL-10/TGF-β regulatory cytokines (23, 64), PD-1/PD-L, and other co-inhibitory molecules such as LAG-3, BTLA-4, CTLA-4, Tim-3, etc. (9, 65). We postulate that synergetic effects from different inhibitory pathways may contribute to *T. spiralis*-induced CD4^+^ T cells hyporesponsiveness besides PD-1/PD-L. Therefore, blocking PD-1/PD-L inhibitory pathway may not take away the whole inhibitory effects induced by *T. spiralis* infection.

In summary, this study demonstrates that *T. spiralis* infection significantly reduced the pathology of CIA in mice by inhibiting Th1/Th17 pro-inflammatory responses and inducing Th2/Treg polarization. PD-1 plays a critical role within the helminth-involved immunomodulation of CD4^+^ T cell subsets which are central mediators of RA. However, the detailed molecular interaction between PD-1/PD-L pathway and the helminth-iTreg cell and cytokine IL-10 and TGF-β still remains unknown. Further studies are needed to explore the mechanism of PD-1-mediated regulation of immune response during helminth infection and autoimmune diseases.

## Ethics Statement

This study was carried out in accordance with the recommendations of “IRB of Capital Medical University.” The protocol was approved by the Animal Care and Use Committee of Capital Medical University (AEEI-2016-008) and comply with the National Institutes of Health Guidelines for the Care and Use of Experimental Animals.

## Author Contributions

YC and XPZ conceived and designed the experiments. YC, XZ, XW, QZ, XH, and JH performed the experiments. YC, XPZ, BZ, and XS analyzed the data. XPZ, YC, and BZ wrote the paper. All authors reviewed the manuscript.

## Conflict of Interest Statement

The authors declare that the research was conducted in the absence of any commercial or financial relationships that could be construed as a potential conflict of interest.
